# Characterization of the complete mitochondrial DNA sequence of the *Ariosoma meeki* (Anguilliformes, Congridae)

**DOI:** 10.1080/23802359.2020.1781557

**Published:** 2020-06-23

**Authors:** Zhi-Qiang Liu, Yao-zhong Shen, Ming Zhao, Wei Wang, Wei Chen, Chun-Yan Ma, Feng-Ying Zhang, Ling-Bo Ma

**Affiliations:** a Key Laboratory of Oceanic and Polar Fisheries, Ministry of Agriculture and Rural Affairs, East China Sea Fisheries Research Institute Chinese Academy of Fishery Sciences, Shanghai, China; b College of Fisheries and Life Sciences, Shanghai Ocean University, Shanghai, China

**Keywords:** *Ariosoma meeki*, Anguilliformes, mitochondrial genome, phylogenetic relationship

## Abstract

In this study, the complete mitochondrial genome of *Ariosoma meeki* was sequenced, assembled and annotated. The circular genome is 16,154 bp in length with nucleotide composition is 28.42% A, 26.53% T, 19.65% G, and 25.40% C and contains 13 protein-coding genes (PCGs), 21 transfer RNA genes (tRNAs), 2 ribosomal RNA unit genes and a large non-coding region (putative control region). To further explore the evolution relationship of the Anguilliformes, we constructed the phylogenetic tree and found that the *A. meeki* had closer relationship with *Ariosoma shiroanago*. This study provided the valuable evidence on phylogenetic relationship of the *A. meeki* at the molecular level and essential resource for further study the molecular phylogenetic, biogeography and adaptive evolution of this lineage.

The true eels, Anguilliformes belongs to Osteichthyes, Actinopterygii, are an ecologically diverse fish that predominantly lives in tropical and subtropical oceans. To date, there are about 800 species have been discovered and they were classified into four suborders according to their morphologic characteristics (Shen et al. 2014). However, existing classification based on morphological data alone has been questioned because of the morphological approach can lead to a confusing justification to distinguish between the individuals (Francesco et al. 2013). Mitochondrial DNA (mtDNA) is effective molecular marker to discriminate the species of one individual because of its conservation (Ladoukakis and Zouros 2017). Therefore, complete mtDNA can be combined with morphological assay to verify the assessment of the Anguilliformes with more-higher resolution.

*A. meeki* is a member of the Anguilliformes, in this study, an adult of *A. meeki* was identified and collected from Chongming, Shanghai Province, China (N31°37′37.42, E121°21′31.01). The specimen, Accession number of which was ARM-20190801, was preserved in Herbarium of Marine animals, East China Sea Fisheries Research Institute, Chinese Academy of Fishery Science and genomic DNA was extracted from muscle tissue by using TIANamp Marine Animal DNA Kit (TIANGEN Biotech, Beijing, China) following the manufacturer’s protocol. After sequencing with the second-generation sequencing technology and assembling with the mitoMaker software, a typical circular mtDNA in size of 16,154 bp was obtained (genbank accession number: MN616974). And its overall base composition is as follows: 28.42% A, 26.53% T, 19.65% G, and 25.40% C. Further, assembled *A. meeki* mitogenome sequence was annotated using the on-line analysis software DOGMA (http://dogma.ccbb.utexas.edu/index.html) and this mtDNA had a conserved structural organization including 13 protein-coding genes (ND1, ND2, ND6, COX1, COII, ATPase 8, ATPase 6, COIII, ND3, ND4L, ND4, ND5 and Cyt b), 21 tRNAs (Phe, Val, Leu, Ile, Gln, Met, Trp, Ala, Asn, Cys, Tyr, Pro, Ser, Asp, Lys, Gly, Arg, His, Ser, Leu, Thr), 2 rRNA genes (12S rRNA and 16S rRNA) and a control region displacement loop (D-loop). All of the 13 protein genes are encoded on the heavy strand and most of them begin with an ATG start codon except for COX1 (started with GTG) and ND6 (started with ATC). The 2 rRNA genes, all encoded on the heavy strand, share similar features in length (955 bp and 1700 bp in size respectively) and sequence characterization with those in other Anguilliformes. Interestingly, unlike other Anguilliformes mtDNA, there are two tRNA-Leu and tRNA-Ser on the *A. meeki* mtDNA, while the tRNA-Glu was not detected (Lü et al. 2019).

To validate the phylogenetic position of *A. meeki*, a Neighbor joining tree was constructed with the complete mtDNA genome sequences of 36 species from the genus Anguilliformes. The mtDNA sequence of *Auxis thazard*, *Tropheus moorii* and *Petrochromis trewavasae* was used as outgroup for tree rooting. The result showed that *A. meeki* is close to *A. shiroanago* and unambiguously grouped with other Anguilliformes species (Figure 1). In conclusion, the newly reported *A. meeki* mtDNA provides essential and important DNA molecular data for further phylogenetic and evolutionary analysis for Anguilliformes.

**Figure 1.**
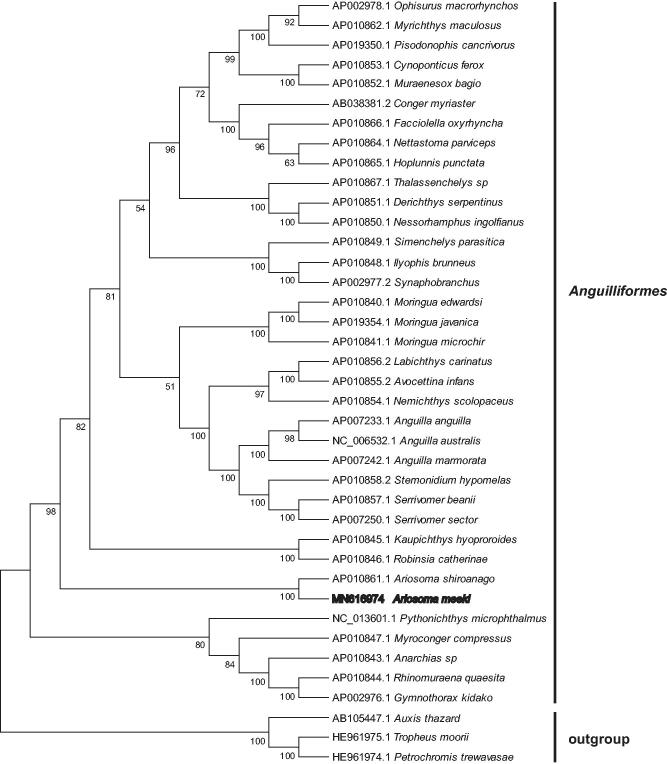
Neighbor joining phylogenetic tree (bootstrap repeat is 10,000) of 36 Anguilliformes and 3 other species (genbank accession number were showed on the left of the species) based on the complete mitochondrial genomes. The numbers above branches indicate bootstrap support values of neighbor joining phylogenetic tree.

## Data Availability

The data that support the findings of this study are openly available in [National Center for Biotechnology Information] at [https://www.ncbi.nlm.nih.gov/nuccore/MN616974.1], reference number [genbank accession number: MN616974].
